# Benzamide–picric acid (1/1)

**DOI:** 10.1107/S1600536810024232

**Published:** 2010-06-26

**Authors:** M. S. Sivaramkumar, R. Velmurugan, M. Sekar, P. Ramesh, M. N. Ponnuswamy

**Affiliations:** a Post Graduate and Research Department of Chemistry, Kongunadu College of Arts and Science, Coimbatore 641 029, India; b Post Graduate and Research Department of Chemistry, Sri Ramakrishna Mission Vidyalaya College of Arts and Science, Coimbatore 641 020, India; c Centre of Advanced Study in Crystallography and Biophysics, University of Madras, Guindy Campus, Chennai 600 025, India

## Abstract

In the title compound, C_7_H_7_NO·C_6_H_3_N_3_O_7_, one of the nitro groups of the picric acid mol­ecule lies in the plane of the attached benzene ring [dihedral angle = 1.4 (1)°] while the other two are twisted away by 9.9 (1) and 30.3 (1)°. In the benzamide mol­ecule, the amide group is almost coplanar with the benzene ring [dihedral angle = 4.4 (1)°]. An intra­molecular O—H⋯O hydrogen bond generates an *S*6 ring motif. In the crystal, mol­ecules are linked into a ribbon-like structure along the *b* axis by O—H⋯O and N—H⋯O inter­molecular hydrogen bonds. In addition, C—H⋯O hydrogen bonds and short O⋯O contacts [2.828 (2) Å] are observed.

## Related literature

For crystal structures of picric acid complexes, see: In *et al.* (1997 ▶); Zaderenko *et al.* (1997 ▶); Nagata *et al.* (1995 ▶); Smith *et al.* (2004 ▶); Goto *et al.* (2004 ▶). For graph-set notation, see: Bernstein *et al.* (1995 ▶).
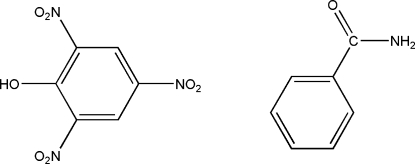


## Experimental

### 

#### Crystal data

C_7_H_7_NO·C_6_H_3_N_3_O_7_
*M* *_r_* = 350.25
Monoclinic, 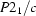

*a* = 7.8644 (3) Å
*b* = 7.0664 (3) Å
*c* = 25.658 (1) Å
β = 90.978 (4)°
*V* = 1425.68 (10) Å^3^
*Z* = 4
Mo *K*α radiation
μ = 0.14 mm^−1^
*T* = 110 K
0.22 × 0.19 × 0.17 mm

#### Data collection

Bruker SMART APEXII area-detector diffractometer
Absorption correction: multi-scan (*SADABS*; Bruker, 2008 ▶) *T*_min_ = 0.970, *T*_max_ = 0.977
8136 measured reflections
3309 independent reflections
2518 reflections with *I* > 2σ(*I*)
*R*_int_ = 0.026

#### Refinement

*R*[*F* ^2^ > 2σ(*F* ^2^)] = 0.040
*wR*(*F* ^2^) = 0.102
*S* = 1.03
3309 reflections
238 parameters
H atoms treated by a mixture of independent and constrained refinement
Δρ_max_ = 0.28 e Å^−3^
Δρ_min_ = −0.27 e Å^−3^

### 

Data collection: *APEX2* (Bruker, 2008 ▶); cell refinement: *SAINT* (Bruker, 2008 ▶); data reduction: *SAINT*; program(s) used to solve structure: *SHELXS97* (Sheldrick, 2008 ▶); program(s) used to refine structure: *SHELXL97* (Sheldrick, 2008 ▶); molecular graphics: *ORTEP-3* (Farrugia, 1997 ▶); software used to prepare material for publication: *SHELXL97* and *PLATON* (Spek, 2009 ▶).

## Supplementary Material

Crystal structure: contains datablocks global, I. DOI: 10.1107/S1600536810024232/ci5096sup1.cif

Structure factors: contains datablocks I. DOI: 10.1107/S1600536810024232/ci5096Isup2.hkl

Additional supplementary materials:  crystallographic information; 3D view; checkCIF report

## Figures and Tables

**Table 1:** Hydrogen-bond geometry (Å, °)

*D*—H⋯*A*	*D*—H	H⋯*A*	*D*⋯*A*	*D*—H⋯*A*
O1—H1⋯O2	0.94 (3)	1.92 (3)	2.6473 (16)	132 (2)
O1—H1⋯O8	0.94 (3)	1.85 (3)	2.5603 (16)	130 (2)
N4—H4*A*⋯O7^i^	0.87 (2)	2.33 (2)	3.120 (2)	150 (2)
N4—H4*B*⋯O8^i^	0.90 (2)	2.08 (2)	2.9702 (19)	167 (2)
C5—H5⋯O6^ii^	0.95	2.39	3.257 (2)	152
C9—H9⋯O4^iii^	0.95	2.50	3.185 (2)	129

Symmetry codes: (i) 
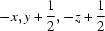
; (ii) 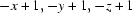; (iii) 
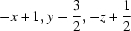
.

**Fig. 1.**
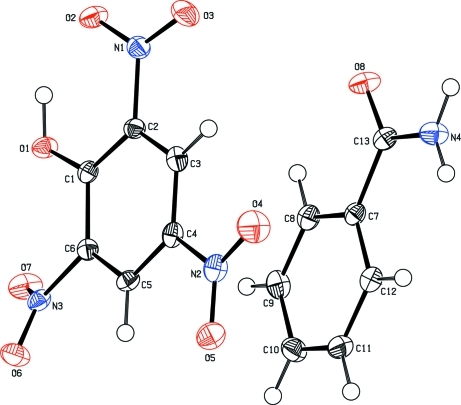
The asymmetric unit of the title compound. Displacement ellipsoids are drawn at the 50% probability level.

**Fig. 2.**
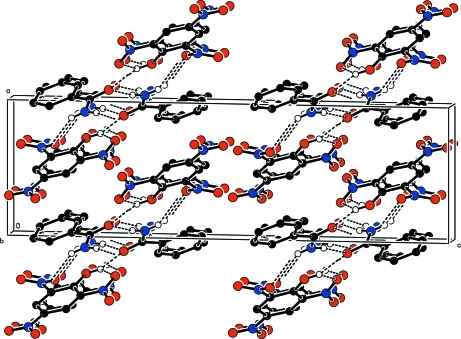
The crystal packing of the title compound, viewed down the b axis.

## supplementary crystallographic information

### Comment

2,4,6-Trinitro phenol, popularly known as picric acid, was primarily used to manufacture explosives and also used as an intermediate in dye manufacturing. It is well known that picric acid forms charge transfer molecular complexes with a number of aromatic compounds such as aromatic hydrocarbons, amines *etc*. through electrostatic or hydrogen bonding interactions (In *et al.*, 1997; Zaderenko *et al.*, 1997). The crystal structures of a large number of picrate salts and picric acid complexes have been studied to understand the conformational features and charge transfer processes (Nagata *et al.*, 1995; Smith *et al.*, 2004; Goto *et al.*, 2004). We report here the crystal structure of the title compound.

In the picric acid molecule (Fig.1), one of the nitro groups lies in the plane of the attached benzene ring and other two rings are twisted away by 9.9 (1)° [N1/O2/O3] and 30.3 (1)° [N3/O6/O7]; the hydroxyl O atom deviates from the attached benzene ring by 0.039 (1) Å. In the benzamide molecule, the amide group is almost coplanar with the benzene ring (C7—C12) [dihedral angle is 4.4 (1)°]. The sum of the bond angles around the atom N4 (359.9°) of the amide group is in accordance with *sp*^2^ hybridization. An intramolecular O1—H1···O2 hydrogen bond forms an S6 ring motif (Bernstein *et al.*, 1995).

The molecules at (*x*, *y*, *z*) and (1-*x*, 1-*y*, 1-*z*) are linked by pairs of C5—H5···O6 intermolecular hydrogen bonds forming a centrosymmetric dimer containing *R*_2_^2^(10) ring motif (Table 1). Atom N4 at (*x*, *y*, *z*) acts as a donor to atom O8 at (-*x*, 1/2 + *y*, 1/2 -*z*) forming a C4 zigzag chain running along the *b* axis. The crystal packing is  controlled by O—H···O, N—H···O and C—H···O types of intermolecular hydrogen bonds, which form a three-dimensional network (Fig.2). An intermolecular O2···O8 short contact of 2.828 (2) Å is observed.

### Experimental

Picric acid (2.29 g) dissolved in methanol was added dropwise to a methanolic solution of benzamide (1.21 g). The solution was stirred at room temperature for 2 h. Single crystals suitable for X-ray analysis are obtained by repeated recrystallization of the salt from pure methanol.

### Refinement

The O- and N-bound H atoms were located in a difference map and refined isotropically. The remaining H atoms were positioned geometrically (C–H = 0.95 Å) and allowed to ride on their parent atoms, with *U*_iso_(H) = 1.2*U*_eq_(C).

### Crystal data

**Table:**

C_7_H_7_NO·C_6_H_3_N_3_O_7_	*F*(000) = 720
*M**_r_* = 350.25	*D*_x_ = 1.632 Mg m^−^^3^
Monoclinic, *P*2_1_/*c*	Mo *K*α radiation, λ = 0.71073 Å
Hall symbol: -P 2ybc	Cell parameters from 1043 reflections
*a* = 7.8644 (3) Å	θ = 3.0–29.2°
*b* = 7.0664 (3) Å	µ = 0.14 mm^−^^1^
*c* = 25.658 (1) Å	*T* = 110 K
β = 90.978 (4)°	Block, colourless
*V* = 1425.68 (10) Å^3^	0.22 × 0.19 × 0.17 mm
*Z* = 4	

### Data collection

**Table:**

Bruker SMART APEXII area-detector diffractometer	3309 independent reflections
Radiation source: fine-focus sealed tube	2518 reflections with *I* > 2σ(*I*)
graphite	*R*_int_ = 0.026
ω and φ scans	θ_max_ = 29.2°, θ_min_ = 3.0°
Absorption correction: multi-scan (*SADABS*; Bruker, 2008)	*h* = −9→10
*T*_min_ = 0.970, *T*_max_ = 0.977	*k* = −9→9
8136 measured reflections	*l* = −33→32

### Refinement

**Table:**

Refinement on *F*^2^	Primary atom site location: structure-invariant direct methods
Least-squares matrix: full	Secondary atom site location: difference Fourier map
*R*[*F*^2^ > 2σ(*F*^2^)] = 0.040	Hydrogen site location: inferred from neighbouring sites
*wR*(*F*^2^) = 0.102	H atoms treated by a mixture of independent and constrained refinement
*S* = 1.02	*w* = 1/[σ^2^(*F*_o_^2^) + (0.0535*P*)^2^ + 0.2776*P*] where *P* = (*F*_o_^2^ + 2*F*_c_^2^)/3
3309 reflections	(Δ/σ)_max_ = 0.011
238 parameters	Δρ_max_ = 0.28 e Å^−^^3^
0 restraints	Δρ_min_ = −0.27 e Å^−^^3^

### Special details

**Table:**

Geometry. All e.s.d.'s (except the e.s.d. in the dihedral angle between two l.s. planes) are estimated using the full covariance matrix. The cell e.s.d.'s are taken into account individually in the estimation of e.s.d.'s in distances, angles and torsion angles; correlations between e.s.d.'s in cell parameters are only used when they are defined by crystal symmetry. An approximate (isotropic) treatment of cell e.s.d.'s is used for estimating e.s.d.'s involving l.s. planes.
Refinement. Refinement of *F*^2^ against ALL reflections. The weighted *R*-factor *wR* and goodness of fit *S* are based on *F*^2^, conventional *R*-factors *R* are based on *F*, with *F* set to zero for negative *F*^2^. The threshold expression of *F*^2^ > σ(*F*^2^) is used only for calculating *R*-factors(gt) *etc*. and is not relevant to the choice of reflections for refinement. *R*-factors based on *F*^2^ are statistically about twice as large as those based on *F*, and *R*- factors based on ALL data will be even larger.

### Fractional atomic coordinates and isotropic or equivalent isotropic displacement parameters (Å^2^)

**Table:**

	*x*	*y*	*z*	*U*_iso_*/*U*_eq_	
O1	0.26955 (15)	0.56145 (16)	0.32065 (4)	0.0186 (3)	
H1	0.234 (3)	0.606 (4)	0.2877 (10)	0.065 (8)*	
O2	0.27599 (15)	0.83691 (16)	0.25079 (4)	0.0217 (3)	
O3	0.45097 (16)	1.06988 (18)	0.26121 (5)	0.0302 (3)	
O4	0.70184 (17)	1.20512 (18)	0.42672 (5)	0.0335 (3)	
O5	0.68930 (15)	0.99973 (18)	0.48912 (4)	0.0252 (3)	
O6	0.32595 (16)	0.46474 (17)	0.46982 (4)	0.0249 (3)	
O7	0.32915 (16)	0.32061 (16)	0.39538 (5)	0.0259 (3)	
N1	0.38210 (17)	0.92648 (19)	0.27671 (5)	0.0179 (3)	
N2	0.65720 (17)	1.0525 (2)	0.44464 (5)	0.0203 (3)	
N3	0.35066 (16)	0.46102 (18)	0.42288 (5)	0.0166 (3)	
C1	0.36450 (19)	0.6822 (2)	0.34693 (6)	0.0144 (3)	
C2	0.4245 (2)	0.8595 (2)	0.32927 (6)	0.0148 (3)	
C3	0.5215 (2)	0.9797 (2)	0.36018 (6)	0.0167 (3)	
H3	0.5615	1.0967	0.3469	0.020*	
C4	0.5587 (2)	0.9257 (2)	0.41060 (6)	0.0163 (3)	
C5	0.5038 (2)	0.7559 (2)	0.43079 (6)	0.0156 (3)	
H5	0.5297	0.7220	0.4659	0.019*	
C6	0.41086 (19)	0.6370 (2)	0.39906 (6)	0.0145 (3)	
O8	0.10176 (16)	0.49021 (16)	0.23654 (4)	0.0223 (3)	
N4	−0.05714 (19)	0.6826 (2)	0.18599 (6)	0.0213 (3)	
H4A	−0.103 (3)	0.705 (3)	0.1554 (9)	0.039 (6)*	
H4B	−0.060 (3)	0.766 (3)	0.2127 (8)	0.034 (6)*	
C7	0.0404 (2)	0.3810 (2)	0.15103 (6)	0.0156 (3)	
C8	0.1221 (2)	0.2114 (2)	0.16244 (6)	0.0180 (3)	
H8	0.1681	0.1906	0.1964	0.022*	
C9	0.1373 (2)	0.0724 (2)	0.12491 (6)	0.0211 (4)	
H9	0.1930	−0.0433	0.1332	0.025*	
C10	0.0710 (2)	0.1023 (2)	0.07508 (6)	0.0231 (4)	
H10	0.0818	0.0072	0.0492	0.028*	
C11	−0.0112 (2)	0.2708 (2)	0.06315 (6)	0.0221 (4)	
H11	−0.0566	0.2910	0.0291	0.026*	
C12	−0.0273 (2)	0.4104 (2)	0.10097 (6)	0.0187 (3)	
H12	−0.0842	0.5255	0.0928	0.022*	
C13	0.0304 (2)	0.5238 (2)	0.19409 (6)	0.0162 (3)	

### Atomic displacement parameters (Å^2^)

**Table:**

	*U*^11^	*U*^22^	*U*^33^	*U*^12^	*U*^13^	*U*^23^
O1	0.0225 (6)	0.0159 (6)	0.0172 (6)	−0.0032 (5)	−0.0053 (4)	0.0010 (4)
O2	0.0253 (6)	0.0198 (6)	0.0198 (6)	−0.0010 (5)	−0.0075 (5)	0.0011 (5)
O3	0.0324 (7)	0.0296 (7)	0.0285 (7)	−0.0124 (6)	−0.0014 (5)	0.0119 (5)
O4	0.0418 (8)	0.0196 (7)	0.0387 (8)	−0.0148 (6)	−0.0071 (6)	0.0005 (5)
O5	0.0269 (7)	0.0276 (7)	0.0210 (6)	−0.0035 (6)	−0.0044 (5)	−0.0051 (5)
O6	0.0320 (7)	0.0247 (7)	0.0180 (6)	−0.0040 (6)	−0.0008 (5)	0.0045 (5)
O7	0.0372 (7)	0.0128 (6)	0.0273 (6)	−0.0005 (6)	−0.0080 (5)	−0.0008 (5)
N1	0.0181 (7)	0.0170 (7)	0.0188 (7)	0.0025 (6)	0.0019 (5)	0.0022 (5)
N2	0.0191 (7)	0.0179 (7)	0.0237 (7)	−0.0022 (6)	−0.0002 (5)	−0.0053 (6)
N3	0.0158 (7)	0.0133 (7)	0.0206 (7)	0.0001 (6)	−0.0046 (5)	0.0014 (5)
C1	0.0127 (7)	0.0139 (7)	0.0167 (7)	0.0022 (7)	0.0003 (6)	−0.0019 (6)
C2	0.0145 (7)	0.0160 (8)	0.0141 (7)	0.0031 (7)	0.0008 (6)	0.0007 (6)
C3	0.0158 (8)	0.0126 (7)	0.0218 (8)	0.0010 (7)	0.0034 (6)	−0.0006 (6)
C4	0.0145 (8)	0.0154 (8)	0.0191 (7)	−0.0014 (7)	0.0003 (6)	−0.0048 (6)
C5	0.0155 (8)	0.0163 (8)	0.0149 (7)	0.0031 (7)	−0.0017 (6)	−0.0015 (6)
C6	0.0145 (8)	0.0117 (7)	0.0174 (7)	0.0017 (6)	0.0009 (6)	0.0004 (6)
O8	0.0302 (7)	0.0170 (6)	0.0196 (6)	−0.0018 (5)	−0.0092 (5)	0.0007 (5)
N4	0.0293 (8)	0.0154 (7)	0.0190 (7)	0.0035 (7)	−0.0040 (6)	−0.0007 (6)
C7	0.0132 (7)	0.0158 (8)	0.0180 (7)	−0.0034 (6)	0.0005 (6)	0.0011 (6)
C8	0.0161 (8)	0.0179 (8)	0.0201 (8)	−0.0014 (7)	0.0006 (6)	0.0030 (6)
C9	0.0214 (8)	0.0161 (8)	0.0260 (9)	0.0015 (7)	0.0042 (7)	0.0023 (6)
C10	0.0228 (9)	0.0230 (9)	0.0237 (8)	−0.0016 (8)	0.0035 (7)	−0.0068 (7)
C11	0.0202 (9)	0.0275 (9)	0.0185 (8)	−0.0009 (8)	−0.0024 (6)	−0.0015 (7)
C12	0.0156 (8)	0.0193 (8)	0.0211 (8)	−0.0002 (7)	−0.0008 (6)	0.0018 (6)
C13	0.0163 (8)	0.0139 (8)	0.0183 (8)	−0.0045 (7)	−0.0008 (6)	0.0024 (6)

### Geometric parameters (Å, °)

**Table:**

O1—C1	1.3126 (19)	C5—H5	0.95
O1—H1	0.94 (3)	O8—C13	1.2399 (19)
O2—N1	1.2330 (17)	N4—C13	1.331 (2)
O3—N1	1.2189 (17)	N4—H4A	0.87 (2)
O4—N2	1.2262 (18)	N4—H4B	0.90 (2)
O5—N2	1.2230 (18)	C7—C8	1.389 (2)
O6—N3	1.2237 (17)	C7—C12	1.397 (2)
O7—N3	1.2275 (17)	C7—C13	1.499 (2)
N1—C2	1.4623 (19)	C8—C9	1.382 (2)
N2—C4	1.464 (2)	C8—H8	0.95
N3—C6	1.468 (2)	C9—C10	1.389 (2)
C1—C2	1.416 (2)	C9—H9	0.95
C1—C6	1.417 (2)	C10—C11	1.387 (2)
C2—C3	1.383 (2)	C10—H10	0.95
C3—C4	1.375 (2)	C11—C12	1.391 (2)
C3—H3	0.95	C11—H11	0.95
C4—C5	1.379 (2)	C12—H12	0.95
C5—C6	1.372 (2)		
			
C1—O1—H1	113.9 (16)	C5—C6—N3	116.37 (13)
O3—N1—O2	123.45 (14)	C1—C6—N3	120.28 (13)
O3—N1—C2	118.32 (13)	C13—N4—H4A	120.1 (14)
O2—N1—C2	118.22 (13)	C13—N4—H4B	116.6 (13)
O5—N2—O4	124.21 (14)	H4A—N4—H4B	123.2 (19)
O5—N2—C4	117.97 (13)	C8—C7—C12	119.32 (15)
O4—N2—C4	117.82 (13)	C8—C7—C13	117.10 (14)
O6—N3—O7	124.09 (14)	C12—C7—C13	123.59 (15)
O6—N3—C6	116.70 (12)	C9—C8—C7	120.77 (15)
O7—N3—C6	119.21 (12)	C9—C8—H8	119.6
O1—C1—C2	126.91 (14)	C7—C8—H8	119.6
O1—C1—C6	118.25 (14)	C8—C9—C10	119.85 (16)
C2—C1—C6	114.82 (13)	C8—C9—H9	120.1
C3—C2—C1	122.92 (14)	C10—C9—H9	120.1
C3—C2—N1	116.43 (14)	C11—C10—C9	120.01 (15)
C1—C2—N1	120.64 (14)	C11—C10—H10	120.0
C4—C3—C2	118.41 (14)	C9—C10—H10	120.0
C4—C3—H3	120.8	C10—C11—C12	120.18 (16)
C2—C3—H3	120.8	C10—C11—H11	119.9
C3—C4—C5	122.10 (14)	C12—C11—H11	119.9
C3—C4—N2	119.57 (14)	C11—C12—C7	119.87 (16)
C5—C4—N2	118.32 (14)	C11—C12—H12	120.1
C6—C5—C4	118.48 (14)	C7—C12—H12	120.1
C6—C5—H5	120.8	O8—C13—N4	121.60 (15)
C4—C5—H5	120.8	O8—C13—C7	119.32 (14)
C5—C6—C1	123.24 (14)	N4—C13—C7	119.08 (14)
			
O1—C1—C2—C3	178.66 (14)	O1—C1—C6—C5	−177.29 (14)
C6—C1—C2—C3	0.3 (2)	C2—C1—C6—C5	1.2 (2)
O1—C1—C2—N1	0.1 (2)	O1—C1—C6—N3	−1.1 (2)
C6—C1—C2—N1	−178.30 (13)	C2—C1—C6—N3	177.36 (13)
O3—N1—C2—C3	9.0 (2)	O6—N3—C6—C5	28.3 (2)
O2—N1—C2—C3	−169.57 (13)	O7—N3—C6—C5	−151.32 (14)
O3—N1—C2—C1	−172.29 (14)	O6—N3—C6—C1	−148.15 (14)
O2—N1—C2—C1	9.1 (2)	O7—N3—C6—C1	32.3 (2)
C1—C2—C3—C4	−1.2 (2)	C12—C7—C8—C9	0.2 (2)
N1—C2—C3—C4	177.46 (13)	C13—C7—C8—C9	−179.82 (14)
C2—C3—C4—C5	0.6 (2)	C7—C8—C9—C10	0.3 (2)
C2—C3—C4—N2	−178.32 (13)	C8—C9—C10—C11	−0.4 (2)
O5—N2—C4—C3	−179.43 (14)	C9—C10—C11—C12	0.1 (2)
O4—N2—C4—C3	0.8 (2)	C10—C11—C12—C7	0.4 (2)
O5—N2—C4—C5	1.6 (2)	C8—C7—C12—C11	−0.5 (2)
O4—N2—C4—C5	−178.23 (14)	C13—C7—C12—C11	179.50 (15)
C3—C4—C5—C6	0.8 (2)	C8—C7—C13—O8	3.8 (2)
N2—C4—C5—C6	179.76 (13)	C12—C7—C13—O8	−176.21 (15)
C4—C5—C6—C1	−1.8 (2)	C8—C7—C13—N4	−175.26 (14)
C4—C5—C6—N3	−178.05 (13)	C12—C7—C13—N4	4.7 (2)

### Hydrogen-bond geometry (Å, °)

**Table:**

*D*—H···*A*	*D*—H	H···*A*	*D*···*A*	*D*—H···*A*
O1—H1···O2	0.94 (3)	1.92 (3)	2.6473 (16)	132 (2)
O1—H1···O8	0.94 (3)	1.85 (3)	2.5603 (16)	130 (2)
N4—H4A···O7^i^	0.87 (2)	2.33 (2)	3.120 (2)	150 (2)
N4—H4B···O8^i^	0.90 (2)	2.08 (2)	2.9702 (19)	167 (2)
C5—H5···O6^ii^	0.95	2.39	3.257 (2)	152
C9—H9···O4^iii^	0.95	2.50	3.185 (2)	129

Symmetry codes:  (i) −*x*, *y*+1/2, −*z*+1/2; (ii) −*x*+1, −*y*+1, −*z*+1; (iii) −*x*+1, *y*−3/2, −*z*+1/2.
